# Intoxication in Children From Opioids Prescribed to Family Members

**DOI:** 10.1001/jamanetworkopen.2026.3515

**Published:** 2026-03-26

**Authors:** Yaron Finkelstein, Szimonetta Komjáthiné Szépligeti, Erzsébet Horváth-Puhó, Lars Pedersen, Stephen B. Freedman, Henrik T. Sørensen, Eyal Cohen

**Affiliations:** 1 Division of Emergency Medicine, Department of Pediatrics, Hospital for Sick Children, University of Toronto, Toronto, Ontario, Canada; 2 Division of Clinical Pharmacology and Toxicology, Department of Pediatrics, Hospital for Sick Children, University of Toronto, Toronto, Ontario, Canada; 3 Child Health Evaluative Sciences, Research Institute, The Hospital for Sick Children, Toronto, Ontario, Canada; 4 Department of Clinical Epidemiology and Center for Population Medicine, Aarhus University and Aarhus University Hospital, Aarhus, Denmark; 5 Division of Pediatric Emergency Medicine, Department of Pediatrics, Cumming School of Medicine, University of Calgary, Calgary, Alberta, Canada; 6 Edwin S. H. Leong Centre for Healthy Children, Hospital for Sick Children, University of Toronto, Toronto, Ontario, Canada; 7 Division of Paediatric Medicine, Department of Paediatrics, Hospital for Sick Children, University of Toronto, Toronto, Ontario, Canada

## Abstract

**Importance:**

Worldwide, 480 000 deaths are attributable to opioids annually. The risk that they pose to children through family members’ prescriptions is unknown.

**Objective:**

To quantify the association between opioid prescriptions dispensed to a household family member and a serious opioid intoxication event (SOE) in a child.

**Design, Setting, and Participants:**

This case-control, population-based study used data from Denmark’s comprehensive nationwide longitudinal prescription and health care registries over a 27-year period from April 1, 1995, to June 30, 2022. Among 3 761 618 Danish residents younger than 20 years, cases were individuals who experienced an SOE. For each case, there were 10 matched population-based controls without a prior SOE. Household family members were individually linked to case and control participants. Statistical analyses were performed between August 2024 and January 2025.

**Exposure:**

An analgesic prescription filled by a household family member within 3 months before a child’s SOE. The study included 3 categories of children whose family members were (1) exposed to prescription opioids, (2) exposed to nonsteroidal anti-inflammatory drugs (NSAIDs), and (3) unexposed.

**Main Outcome and Measures:**

SOE, defined as death, hospitalization, or emergency department visit due to opioid intoxication. The incidence rate ratio of SOEs among children whose family members redeemed an opioid prescription was compared with those in unexposed households and with those whose family members filled NSAID prescriptions. Conditional logistic regression was used to compute adjusted odds ratios (AORs), controlling for potential sociodemographic and mental health confounders. Subsequently, analyses were stratified by sex, age brackets (<13 vs ≥13 years), SOE calendar year, and hospital disposition. Finally, a sensitivity analysis was conducted restricting the interval between prescription dispensing and the SOE to 1 month.

**Results:**

A total of 2000 children (median age at SOE, 17.8 years [IQR, 15.7-19.0 years]; 1096 boys [54.8%]) experienced an SOE and were matched with 19 840 controls (median age at index, 17.7 years [IQR, 15.6-19.0 years]; 10 872 boys [54.8%]). Among 2000 cases, 1116 (55.8%) were hospitalized and 60 (3.0%) died. Having a family member fill an opioid prescription (319 cases and 1137 controls) was associated with increased odds of an SOE compared with unexposed children (1398 cases and 16 181 controls; AOR, 2.87; 95% CI, 2.45-3.38) and with children exposed to NSAIDs (283 cases and 2522 controls; AOR, 2.22; 95% CI, 1.81-2.72). Odds of an opioid-related death were nearly 4-fold higher compared with unexposed children (15 cases and 33 controls vs 42 cases and 496 controls, respectively; AOR, 3.70; 95% CI, 1.55-8.84). The association remained robust in stratified and sensitivity analyses.

**Conclusions and Relevance:**

This study suggests that children of family members prescribed opioids had increased odds of opioid intoxication–related death and other SOEs compared with children of unexposed families or those exposed to nonopioid analgesics. Public health strategies to mitigate the opioid crisis should consider unique pediatric aspects that can reduce the likelihood of pediatric SOEs.

## Key Points

**Question:**

Are children living in households of family members who recently filled an opioid prescription at increased risk of opioid-related death and other serious opioid events (SOEs)?

**Findings:**

In this 27-year, population-based case-control study of 3 761 618 children in Denmark, the odds were nearly 4-fold higher of opioid-related death and nearly 3-fold higher of any SOE among children whose family members filled an opioid prescription in the prior 3 months compared with children in unexposed households, and the odds were also higher compared with children exposed to nonopioid analgesics.

**Meaning:**

This study suggests that opioids prescribed to household family members are associated with a markedly increased risk of fatal and nonfatal opioid intoxication in children; mitigation strategies may include improved prescribing stewardship and caregiver education to reduce pediatric intoxication.

## Teaser

This case-control study of more than 3 million Danish residents quantifies the association between opioid prescriptions dispensed to a household family member and a serious opioid intoxication event in a child.

## Introduction

Opioids are pivotal in treating severe acute pain.^1^ However, over the past 3 decades, misuse of prescription and illicit opioids has precipitated an “opioid crisis” characterized by dramatic increases in addiction, overdoses, and fatalities. Globally, there are approximately 480 000 opioid-related deaths annually,^2^ and opioids remain the class of medications most commonly associated with fatalities.^3^

The opioid crisis has profoundly impacted children. A study of fatal pediatric intoxications in the US between 2005 and 2018 reported that 47.3% of deaths were attributed to opioids.^4^ A nationwide US study examining pediatric opioid-related fatalities between 2004 and 2020 found that 65.3% occurred in the child’s own residence, with 91.8% attributed to prescription opioids, emphasizing their central role in fatal pediatric overdoses.^5^ Furthermore, the severity of opioid intoxications reported to US poison control centers between 2016 and 2023 increased over the study period,^6^ with a greater proportion resulting in intensive care unit (ICU) admissions.^7^ Similar trends have been observed in Canada,^8^ the UK,^9^ Australia,^10^ Denmark,^11^ Norway, and Sweden.^12^

Widespread opioid prescribing has led to their presence in many homes. Consequently, children and adolescents who are not prescribed opioids may be exposed to opioids unintentionally,^13,14^ through neglect,^15^ or by intentional consumption.^16^ Population-level data on the effect of the opioid crisis on children are limited, and this issue receives little attention in policy initiatives.^13,17^ The downstream effects of prescription opioid dispensing to family members on the children residing in the household remain poorly understood.^13^

To address this gap, we used Denmark’s comprehensive nationwide longitudinal prescription and health care registries to quantify the association between opioid prescription dispensed to a family member and death, hospitalization, or emergency department (ED) visit due to opioid intoxication in household children.

## Methods

### Design and Setting

We conducted a population-based case-control study that included all Danish residents younger than 20 years (cumulative source population: 3 761 618).^18,19^ The study period spanned April 1, 1995, to June 30, 2022. The Danish health care system provides universal tax-supported health care to all Danish citizens and legal residents and guarantees unfettered access to general practitioners, specialists in private practice, hospitals, and universal drug coverage.^18^ This study was exempt from ethics approval and has been reported to the Danish Data Protection Agency; according to Danish legislation, informed consent and approval from an ethics committee are not required for registry-based studies. Data handling procedures complied with Statistics Denmark’s data confidentiality policy. The study adheres to the the Reporting of Studies Conducted Using Observational Routinely-Collected Data for Pharmacoepidemiology (RECORD-PE) guideline.^20^

### Data Sources and Linkage

We used Danish health care,^21^ administrative, and social registries,^22,23,24^ which were linked at the individual level using encrypted personal identifiers. The registries that provided data also include the Danish Civil Registration System, which assigns a unique personal identifier to all Danish residents at birth or on immigration, enabling individual-level linkage across data sources.^25^ This database includes demographic data (eg, age) and information on kinship, thereby permitting the linkage of children with their parents and siblings.^26^ In the Civil Registration System, parenthood is based on legal relationships; thus, adopted children are linked with their legal guardians.^27^ The Danish Medical Birth Registry includes data on all births.^28^ This registry contains information on the index pregnancy (including the parents’ personal identification numbers), maternal pregnancy-related characteristics (eg, parity and pregnancy-related complications), and newborn outcome characteristics (eg, Apgar score and birth weight). The Danish National Prescription Registry captures all medications dispensed by pharmacies across the country.^29^ In this registry, claims are categorized according to the World Health Organization’s Anatomical Therapeutic Chemical classification system. Danish residents receive their medications, including over-the-counter medications, free of charge or with partial reimbursement. The Danish National Patient Registry^30^ includes all diagnoses from individual hospital inpatient admissions, outpatient clinic visits, and ED visits. Diagnostic coding has been based on the *International Statistical Classification of Diseases and Related Health Problems, Tenth Revision* (*ICD-10*) since 1994.^30,31^ The Danish Psychiatric Central Research Register contains data on individuals treated at psychiatric departments.^32^ It captures all psychiatric and substance misuse disorder diagnoses and treatments and relevant demographic information. The Danish Register of Causes of Death compiles information on causes of death, capturing both in-hospital and out-of-hospital deaths. All deaths identified during the study, including opioid-related deaths, were identified from this registry. The Population Income Register, the Population Education Register, and the Integrated Database for Labour Market Research provided information on mothers’ and fathers’ income, level of education, and employment status, respectively.

### Cases and Controls

Cases were defined as patients younger than 20 years with a first serious opioid intoxication event (SOE) within the study period, based on date of ED visit, hospitalization, or opioid-related death. This date served as the index date. We used the Danish National Patient Registry^21^ and the Cause of Death Registry to identify cases meeting SOE criteria based on relevant *ICD-10* codes (eTable 1 in Supplement 1). For each case, we randomly selected up to 10 controls from the general population using the Danish Civil Registration System. Incidence density sampling was performed to select controls who were alive and without a previous SOE. Controls were matched with their respective cases based on age (same birth year), sex, and calendar year of SOE. As controls lacked an event index date, their assigned index date was the same as that of their corresponding case. Household family members (ie, parents and siblings) were linked with the case and control individuals using the Medical Birth Registry and Civil Registration System based on their unique personal identification numbers.

### Exposures: Opioids, NSAIDs, and No Exposure

**Figure 1.**
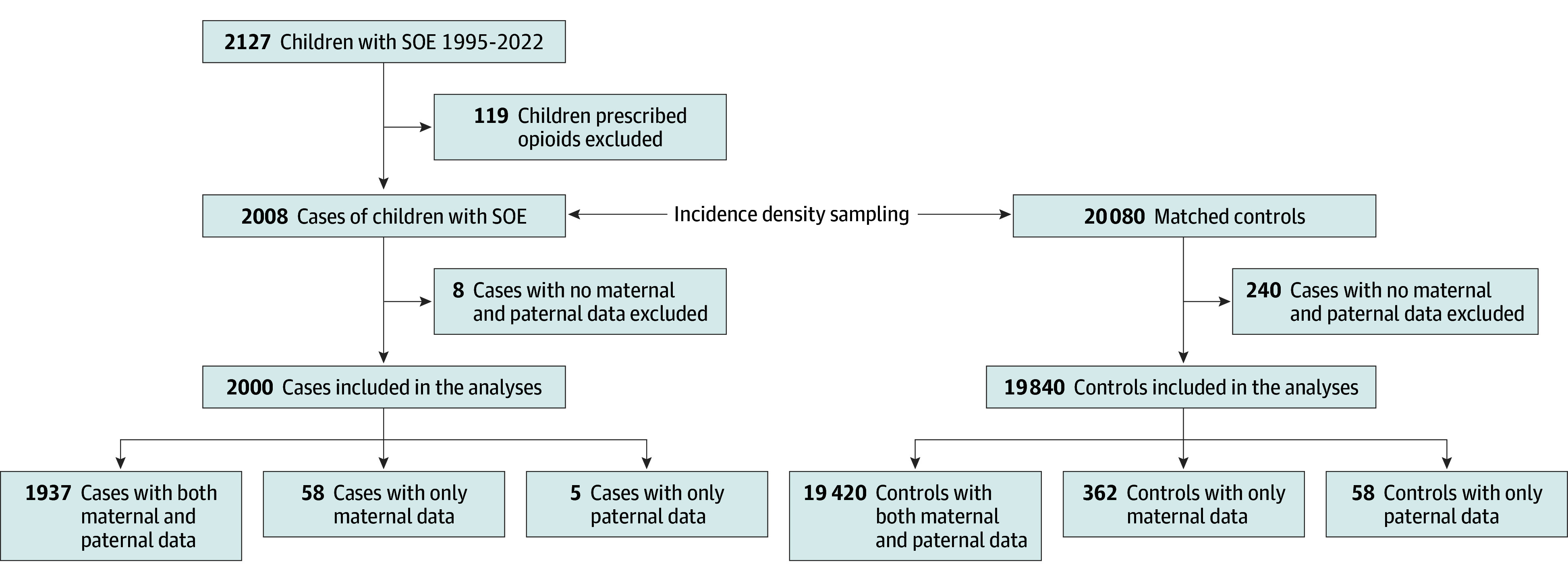
Study Flowchart Illustrating the Case and Control Groups SOE indicates serious opioid event.

Opioid and nonsteroidal anti-inflammatory drug (NSAID) exposures were deemed present for children with a family member who had filled an opioid or NSAID prescription, respectively, within the 3 months preceding the index date. NSAID exposure, defined in a similar way, was selected to represent an alternative control group because, like opioids, NSAIDs are frequently prescribed to treat acute and chronic pain. In Denmark, nonaspirin NSAID use is prescription based (with the exception of low-dose ibuprofen), and their dispensing is captured in the Danish National Prescription Registry,^33^ whereas acetaminophen is sold exclusively over the counter. Families with members who are prescribed analgesics, such as NSAIDs or opioids, may be inherently different from those who are not, and thus they may expose youths to a different household environment. Children whose family members had filled both an opioid and an NSAID prescription were classified as opioid exposed. We considered children to be unexposed if neither their parents nor siblings had filled any opioid or NSAID prescription in the 3 months preceding their index date. Last, we excluded children who had themselves filled a prescription for an opioid or NSAID in the 3 months prior to their index date, as well as case and control children who could not be linked with at least 1 parent (Figure 1).

### Covariates

Child and parental demographic covariates were obtained from the Civil Registration System. We collected indicators of parental socioeconomic status (ie, mother’s and father’s level of education [basic, high school or similar, high], income [low, intermediate, high, very high], and employment status [employed, unemployed, early retirement, state pension]) from the Income Statistics Register, the Population Education Register, and the Integrated Database for Labour Market Research, respectively, using the status assigned for the year preceding the index date. Parent and child substance-related and mental health disorders (eTable 1 in Supplement 1) were ascertained as dichotomous variables from the Danish Psychiatric Central Research Register using a 5-year lookback window (or a lookback window to 1995 for patients included before 2000).^32^ Substance-related disorders, when present, were attributed to children aged 13 years or older.

### Statistical Analysis

Statistical analysis was conducted between August 2024 and January 2025. Incidence of first-time SOE events, along with 95% CIs, were calculated annually by dividing the number of SOEs among Danish residents younger than 20 years by the population at risk (ie, the total number of residents <20 years in each calendar year). We analyzed the data first by creating contingency tables for the main study variables. Second, we performed a conditional logistic regression to calculate the unadjusted odds ratio (OR) and adjusted OR (AOR) with 95% CIs for the association between SOE and presence of a family member who filled (1) an opioid prescription vs no exposure (no opioid and no NSAID) or (2) an opioid vs an NSAID prescription. Controls were sampled from the source population throughout the risk period using density sampling. With this sampling technique, the OR estimates the incidence rate ratio from the corresponding cohort study.^34,35^ We controlled for the matching variables (ie, age, sex, and calendar year) by design. In the adjusted regression models, the following potential confounders, defined a priori, were also included: child’s mental health disorder, child’s substance-related disorder, either parent with a mental health or substance misuse disorder, and parental marital status. We also calculated and compared the incidence rate ratios of SOEs between the studied groups based on event outcome (ie, ED discharge, hospital admission, ICU admission, or opioid-related death). Next, for children whose family members were prescribed opioids, we stratified the analysis by sex, child’s age (<13 vs ≥13 years), and calendar year of SOE or index date. To test the robustness of our findings, we conducted a sensitivity analysis in which the time gap between an opioid or NSAID prescription filled by a family member and an SOE was shortened from 3 months to 1 month and extended to 6 months and 1 year. Given that it is still possible that a small number of children younger than 13 years with opioid intoxication intentionally misused opioids, we conducted a sensitivity analysis with a more conservative definition, using an age cutoff of 10 years. To address potential imbalances in socioeconomic position, we conducted a sensitivity analysis in which the main regression models were repeated with additional adjustment for maternal education, which was used as a proxy for socioeconomic status. Last, an additional sensitivity analysis was carried out after removing intentional events from the analysis. All statistical analyses for the study were performed between August 2024 and January 2025. For analyses we used SAS, version 9.4 software (SAS Institute Inc) and R, version 4.4.2 (R Project for Statistical Computing).

## Results

We identified 2000 patients (median age at SOE, 17.8 years [IQR, 15.7-19.0 years]; 1096 boys [54.8%] and 904 girls [45.2%]) younger than 20 years with an SOE between April 1, 1995, and June 30, 2022 (Table 1). Among these SOE cases, 1116 (55.8%) were admitted to the hospital, 824 (41.2%) were discharged from the ED, and 60 (3.0%) died of opioid intoxication. The 2000 patients with an SOE were matched with 19 840 population controls (median age at index, 17.7 years [IQR, 15.6-19.0 years]; 10 872 boys [54.8%] and 8968 girls [45.2%]) who did not experience an SOE prior to their index date (Figure 1). The demographic characteristics and drug exposures of cases, controls, and their family members are presented in Table 1. Among cases, 276 (13.8%) were younger than 5 years. Compared with controls’ parents, cases’ parents (mother and/or father) were less likely to be in marital or partnered relationships, be employed, or to have postsecondary education. They were more likely to have a lower income and to have received a diagnosis of a substance-related or other mental health disorder. The most common opioid prescription filled by a family member in SOE cases was for tramadol, followed by morphine and oxycodone.

**Table 1.** Characteristics of Children With or Without SOEs, Denmark, 1995-2022

Characteristic	No. (%)	
	SOE cases (n = 2000)	Controls (n = 19 840)
Exposure		
Exposed to family member’s prescription opioids	319 (16.0)	1137 (5.7)
Exposed to family member’s prescription NSAIDs	283 (14.2)	2522 (12.7)
Nonexposed	1398 (69.9)	16 181 (81.6)
Family members with an opioid prescription		
Mother	190 (9.5)	615 (3.1)
Father	149 (7.4)	501 (2.5)
Sibling	18 (1.0)	67 (0.3)
Child’s sex		
Male	1096 (54.8)	10 872 (54.8)
Female	904 (45.2)	8968 (45.2)
Child’s age at SOE or index date, y		
<5	276 (13.8)	2747 (13.8)
5 to <9	25 (1.3)	248 (1.3)
9 to <13	22 (1.1)	268 (1.4)
13 to <17	456 (22.8)	4598 (23.2)
17 to <20	1221 (61.1)	11 979 (60.4)
Calendar year of SOE or index date		
1995-2004	662 (33.1)	6563 (33.1)
2005-2014	917 (45.9)	9105 (45.9)
2015-2022	421 (21.1)	4172 (21.0)
Marital status of parents		
Married or registered partnership	960 (48.0)	13 592 (68.5)
Single	446 (22.3)	2678 (13.5)
Divorced or widowed	582 (29.1)	3445 (17.4)
Missing	7 (0.4)	67 (0.3)
Parental mental health		
Substance-related disorders among mothers	86 (4.3)	193 (1.0)
Substance-related disorders among mothers—missing	5 (0.3)	58 (0.3)
Substance-related disorders among fathers	126 (6.3)	352 (1.8)
Substance-related disorders among fathers—missing	58 (2.9)	365 (1.8)
Mental health disorders other than substance-related among mothers	234 (11.7)	1011 (5.1)
Mental health disorders other than substance-related among mothers—missing	5 (0.3)	58 (0.3)
Mental health disorders other than substance-related among fathers	150 (7.5)	650 (3.3)
Mental health disorders other than substance-related among fathers—missing	58 (2.9)	365 (1.8)
Parental income		
Mother		
Low income	804 (40.2)	5600 (28.2)
Intermediate income	613 (30.7)	6148 (31.0)
High income	362 (18.1)	4874 (24.6)
Very high income	177 (8.9)	2837 (14.3)
Missing information about income	39 (2.0)	323 (1.6)
Father		
Low income	645 (32.3)	3964 (20.0)
Intermediate income	366 (18.3)	3435 (17.3)
High income	430 (21.5)	4706 (23.7)
Very high income	386 (19.3)	6754 (34.0)
Missing information about income	115 (5.8)	616 (3.1)
Parental highest achieved education		
Mother		
Basic education	808 (40.4)	4507 (22.7)
Youth education, high school, or similar	700 (35.0)	7614 (38.4)
High education	379 (19.0)	6705 (33.8)
Unknown educational level	108 (5.4)	956 (4.8)
Father		
Basic education	666 (33.3)	4206 (21.2)
Youth education, high school, or similar	766 (38.3)	8634 (43.5)
Higher education	281 (14.1)	5255 (26.5)
Unknown educational level	229 (11.5)	1380 (7.0)
Parental employment status		
Mother		
Employed	1251 (62.6)	15 567 (78.5)
Unemployed	292 (14.6)	1839 (9.3)
Early retirement	389 (19.5)	1861 (9.4)
State pension	14 (0.7)	46 (0.2)
Unknown	49 (2.5)	469 (2.4)
Father		
Employed	1293 (64.7)	16 013 (80.7)
Unemployed	173 (8.7)	1171 (5.9)
Early retirement	299 (15.0)	1283 (6.5)
State pension	23 (1.2)	116 (0.6)
Unknown	154 (7.7)	892 (4.5)
Mental health of children		
Substance-related disorders	493 (24.7)	291 (1.5)
Mood and affective disorders	105 (5.3)	136 (0.7)
Anxiety and stress disorders	56 (2.8)	155 (0.8)
Schizophrenia or other psychotic disorder	64 (3.2)	59 (0.3)
Other psychiatric disorders	479 (24.0)	788 (4.0)
SOE occurred intentionally (only among cases)	57 (2.9)	NA

Abbreviations: NA, not applicable; NSAIDs, nonsteroidal anti-inflammatory drugs; SOE, serious opioid event.

### Association Between Drug Exposure and SOE

**Figure 2.**
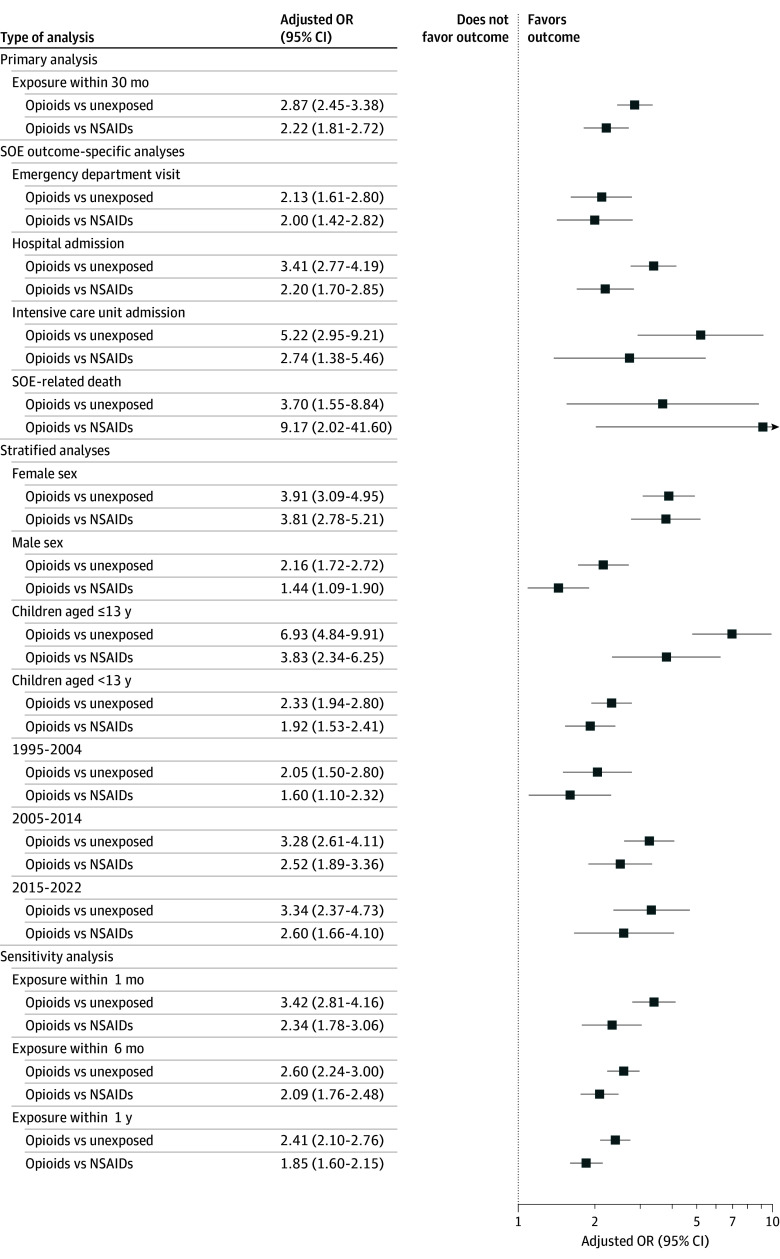
Forest Plot of Serious Opioid Events (SOEs) Among Children: Family Member Opioid Exposure vs Unexposed or Nonsteroidal Anti-Inflammatory Drug (NSAID) Exposed Forest plot illustrating adjusted odds ratios (ORs) of SOEs among children whose family members were prescribed opioids in the preceding 3 months vs unexposed children, or opioid exposed vs NSAID exposed.

Compared with children from households with unexposed family members (1398 cases and 16 181 controls), children in households where an opioid prescription was filled by a family member within the 3 months preceding the index date (319 cases and 1137 controls) were more likely to experience an SOE (AOR, 2.87; 95% CI, 2.45-3.38) (Table 2; Figure 2). Compared with children in households with unexposed family members, exposed children were more likely to be admitted to a hospital ward (unexposed, 747 cases and 9039 controls vs exposed, 192 cases and 654 controls; AOR, 3.41; 95% CI, 2.77-4.19) or an ICU (unexposed, 105 cases and 1315 controls vs exposed, 26 cases and 88 controls; AOR, 5.22; 95% CI, 2.95-9.21) for an SOE. Children living in households with family members prescribed opioids had a nearly 4-fold higher odds of opioid-related death compared with those in unexposed households (exposed, 15 cases and 33 controls vs unexposed, 42 cases and 496 controls; AOR, 3.70; 95% CI, 1.55-8.84) (Figure 2). Last, the odds of an SOE, as well as hospitalization, ICU admission, and opioid-related death, were all greater among children with family members who filled opioid prescriptions vs those who filled NSAID prescriptions in the 3 months preceding the SOE (opioids, 319 cases and 1137 controls vs NSAIDs, 283 cases and 2522 controls; AOR, 2.22; 95% CI, 1.81-2.72) (Table 2; Figure 2). The number of deaths in the NSAID group was small (≤5), resulting in wide 95% CIs for NSAID exposure and substantial overlap with the 95% CIs for opioid-related deaths.

**Table 2.** Odds Ratios of SOEs According to Exposure Groups and Stratified by Sex, Age Group, and Calendar Period

Category and exposure	No.		Odds ratio (95% CI)	
	SOE cases	Controls	Unadjusted	Adjusted
Overall				
Unexposed	1398	16 181	1 [Reference]	1 [Reference]
Opioid prescription	319	1137	3.29 (2.87-3.77)	2.87 (2.45-3.38)
NSAID prescription	283	2522	1 [Reference]	1 [Reference]
Opioid prescription	319	1137	2.53 (2.12-3.02)	2.22 (1.81-2.72)
**Child sex**				
Girls				
Unexposed	619	7283	1 [Reference]	1 [Reference]
Opioid prescription	179	515	4.20 (3.46-5.09)	3.91 (3.09-4.95)
NSAID prescription	106	1170	1 [Reference]	1 [Reference]
Opioid prescription	179	515	3.92 (3.01-5.11)	3.81 (2.78-5.21)
Boys				
Unexposed	779	8898	1 [Reference]	1 [Reference]
Opioid prescription	140	622	2.59 (2.12-3.15)	2.16 (1.72-2.72)
NSAID prescription	177	1352	1 [Reference]	1 [Reference]
Opioid prescription	140	622	1.73 (1.36-2.20)	1.44 (1.09-1.90)
**Child age**				
Aged <13 y				
Unexposed	223	2881	1 [Reference]	1 [Reference]
Opioid prescription	65	109	8.02 (5.66-11.4)	6.93 (4.84-9.91)
NSAID prescription	34	229	1 [Reference]	1 [Reference]
Opioid prescription	65	109	4.17 (2.58-6.75)	3.83 (2.34-6.25)
Aged ≥13 y				
Unexposed	1175	13 300	1 [Reference]	1 [Reference]
Opioid prescription	254	1028	2.82 (2.43-3.28)	2.33 (1.94-2.80)
NSAID prescription	249	2293	1 [Reference]	1 [Reference]
Opioid prescription	254	1028	2.30 (1.90-2.78)	1.92 (1.53-2.41)
**Calendar period of SOE or index date**				
1995-2004				
Unexposed	486	5355	1 [Reference]	1 [Reference]
Opioid prescription	80	356	2.50 (1.93-3.25)	2.05 (1.50-2.80)
NSAID prescription	96	852	1 [Reference]	1 [Reference]
Opioid prescription	80	356	2.00 (1.45-2.76)	1.60 (1.10-2.32)
2005-2014				
Unexposed	612	7350	1 [Reference]	1 [Reference]
Opioid prescription	170	570	3.64 (3.00-4.41)	3.28 (2.61-4.11)
NSAID prescription	135	1185	1 [Reference]	1 [Reference]
Opioid prescription	170	570	2.65 (2.07-3.40)	2.52 (1.89-3.36)
2015-2022				
Unexposed	300	3476	1 [Reference]	1 [Reference]
Opioid prescription	69	211	3.84 (2.84-5.18)	3.34 (2.37-4.73)
NSAID prescription	52	485	1 [Reference]	1 [Reference]
Opioid prescription	69	211	3.13 (2.10-4.67)	2.60 (1.66-4.10)

Abbreviations: NSAID, nonsteroidal anti-inflammatory drug; SOE, serious opioid event.

### Stratified Analyses

In stratified analyses, differences in AORs of SOEs among those exposed to a family member’s opioid prescriptions vs unexposed were particularly marked among girls (exposed, 179 cases and 515 controls vs unexposed, 619 cases and 7283 controls; AOR, 3.91; 95% CI, 3.09-4.95]) and among those younger than 13 years (exposed, 65 cases and 109 controls vs unexposed, 223 cases and 2881 controls; AOR, 6.93; 95% CI, 4.84-9.91) (Table 2; Figure 2). A similar pattern of increased effect size of SOE among girls compared with boys and among those younger than 13 years compared with those aged 13 years or older was observed when the comparison group was those exposed to NSAID prescriptions filled by a family member (Table 2; Figure 2).

### Sensitivity Analysis

In the sensitivity analysis, an increased likelihood of an SOE persisted when the time interval between a dispensed prescription for opioids by a family member and the SOE was restricted to 1 month, whether the comparison group was unexposed children (AOR, 3.42; 95% CI, 2.81-4.16; exposed, 210 cases and 611 controls; unexposed, 1641 cases and 18 088 controls) or an NSAID-exposed control group (149 cases and 1141 controls; AOR, 2.34; 95% CI, 1.78-3.06) (Figure 2). When the exposure window was lengthened to 6 and 12 months, findings remained consistent with the primary analysis (Figure 2). In a sensitivity analysis using a cutoff age of 10 years instead of 13 years, similar findings were observed (eResults in Supplement 1). In the sensitivity analysis with additional adjustment for maternal level of education, the OR estimates remained consistent with the main analyses, albeit slightly attenuated (eTable 2 in Supplement 1). Last, excluding the 2.9% of intentional events (57 of 2000) (Table 1), the results remained consistent with the primary analysis (opioid-exposed vs unexposed children: AOR, 2.75; 95% CI, 2.33-3.24; opioid-exposed vs NSAID-exposed controls: AOR, 2.13; 95% CI, 1.73-2.62).

## Discussion

In this 27-year population-based case-control study, after adjusting for potential confounders, we found that compared with matched unexposed controls, children living in households with family members who filled an opioid prescription were at a markedly elevated risk of experiencing opioid-related severe adverse events. They were nearly 3 times more likely to be hospitalized and nearly 4 times more likely to die from an opioid intoxication event within 3 months of filling the prescription. These increased odds of experiencing serious harm from opioids were particularly pronounced among girls and younger children.

In our study, the parents of children involved in opioid intoxication were more likely to be socially disadvantaged (ie, have lower income, lower level of education, and lower likelihood of being employed) or to be in formal relationships, while they were more likely to have mental health and substance-related disorders. The associations between social vulnerability, opioid prescribing, and intoxication risk among children in the household are complex and multifaceted.^36^ A prior study demonstrated an association between social vulnerability, higher parental opioid prescription rates, and increased child removal by protective services.^37^

Other studies have reported an elevated risk of SOEs among opioid-exposed children, but without assessing the specific risk to children associated with prescription opioids dispensed to all other household members. An earlier Canadian study^38^ restricted to mothers receiving social welfare supports reported almost double the risk of intoxication among young children of mothers who were prescribed opioids. A study of opioid-related mortality rates among individuals 20 years or younger based on Centers for Disease Control and Prevention (CDC) data reported a 268% increase over an 18-year period.^39^ However, the CDC study reported deaths from all opioid sources, and the relative contribution of prescription opioids was unreported. Other previous research has been limited by selective pediatric populations with small sample size,^38^ missing data on kinship,^13^ or restrictive scope (eg, inpatients only^40^ or individuals covered by a specific medical insurer [eg, Medicare^41^ only]).

Access to prescribed “leftover” opioids in households has fueled pediatric opioid exposures.^42^ Up to 90% of patients prescribed opioids after surgery have unused doses, which may be ingested by children.^13,14^ In the US, 41.8% of opioid prescriptions to opioid-naive patients exceeded a 3-day supply, and 3.8% exceeded a 7-day supply.^43^ A study of opioid prescribing at hospital discharge found that prescribed amounts frequently exceeded patient needs.^44^ In addition, only 19% of families were instructed on how to properly dispose of leftover opioids, with only 4% following the instructions provided.^44^ Leftover opioids are commonly stored in unlocked locations (68%), with forgetting cited as the primary reason for not discarding them.^45^ Parental education regarding proper handling, secure storage, and prompt disposal of leftover opioids plays an important role in the prevention of exposures and involves relatively simple and inexpensive steps to reduce the availability of prescription opioids in the home, and it should be a cornerstone of preventing pediatric opioid poisonings. A 2024 study from the US analyzed 107 597 pediatric surgical procedures and found that 59.1% of opioids prescribed were concentrated in 3 procedures.^46^ Targeting opioid stewardship initiatives at clinicians performing these procedures may help curb opioid-related risks.

### Strengths and Limitations

Our study has some strengths, one of which is the inclusion of an entire nation’s population. This enabled us to provide a robust quantification of the magnitude of the problem over almost 3 decades. Discharge diagnoses and dispensed medication data extracted from Danish registries are highly reliable.^21,29^ Another study strength is the generalizability of our findings to North America and other high-income countries. A 2021 study reported similar levels of opioid consumption across Denmark, the US, Canada, Germany, and Spain; in each country, the distribution of daily prescribed morphine milligram equivalents in 2019 was approximately 1000 mg per 1000 residents.^47^

Our study also has some limitations. We relied on linked data from registries, but we did not have access to patients’ medical records and thus could not explore the specific circumstances nor volition (accidental vs intentional) of exposure associated with an SOE in individual cases. However, the sensitivity analysis with an age cutoff of 10 years, which likely better isolates accidental cases (few children aged <10 years misuse opioids intentionally) yielded similar results. Second, although we were able to link parents and siblings, it is possible that some occasional unique household arrangements (eg, household grandparents) were not captured. Third, we do not have information on exposure of children to illicit opioid sources, nor to opioid exposure doses in intoxication events. Future research should explore the implications of fentanyl, which was not a common exposure in our cohort, and may be more dominant in other jurisdictions. Results of the stratified analyses should be considered exploratory and hypothesis generating. Fourth, although we adjusted for a broad set of potential important confounders, unmeasured confounding may still be present.

## Conclusion

In this case-control study, children in the home of family members prescribed opioids had markedly increased odds of opioid intoxication–related death and other severe events leading to hospital-based care compared with children of unexposed families and those exposed to nonopioid analgesics. Public health strategies to mitigate the opioid crisis should consider unique pediatric aspects that can reduce the likelihood of pediatric SOEs. Enhanced safeguards by practitioners, regulators, and caregivers are required when prescribing opioids to patients with children in the household. Effective tools may include clinicians’ attention to prescribing smaller, clinically necessary amounts, blister packaging, surgeon-driven opioid stewardship,^46^ and educational initiatives targeting parents and high-volume procedures and prescribers.^43^

## Notes

Supplement 1. **eTable 1.** International Classification of Diseases, Tenth Edition (ICD-10) Diagnosis Codes and Anatomical Therapeutic Chemical (ATC) Medication Codes Used in the Study **eResults.** Adjusted Odds Ratios for Serious Opioid Events With a Cutoff Age 10 Years **eTable 2.** Sensitivity Analysis Incorporating Maternal Education as a Proxy for Socioeconomic Position in Models of Serious Opioid Events

Supplement 2.
Data Sharing Statement
