# Supplementary material for: Intoxication in Children From Opioids Prescribed to Family Members
Source: JAMA Netw Open. 2026 Mar 26;9(3):e263515. doi: 10.1001/jamanetworkopen.2026.3515 (PMC13022737; doi:10.1001/jamanetworkopen.2026.3515)
Supplement: Supplement 1. — eTable 1. International Classification of Diseases, Tenth Edition (ICD-10) Diagnosis Codes and Anatomical Therapeutic Chemical (ATC) Medication Codes Used in the Study eResults. Adjusted Odds Ratios for Serious Opioid Events With a Cutoff Age 10 Years eTable 2. Sensitivity Analysis Incorporating Maternal Education as a Proxy for Socioeconomic Position in Models of Serious Opioid Events [file jamanetwopen-e263515-s001.pdf]

Supplementary Online Content

Finkelstein Y, Komjáthiné Szépligeti S, Horváth-Puhó E, et al. Intoxication in children from opioids prescribed to family members. *JAMA Netw Open.* 2026;9(3):e263515. doi:10.1001/jamanetworkopen.2026.3515

eTable 1. *International Classification of Diseases, Tenth Edition* (*ICD-10*) Diagnosis Codes and *Anatomical Therapeutic Chemical* (*ATC*) Medication Codes Used in the Study

eResults. Adjusted Odds Ratios for Serious Opioid Events With a Cutoff Age 10 Years

eTable 2. Sensitivity Analysis Incorporating Maternal Education as a Proxy for Socioeconomic Position in Models of Serious Opioid Events

This supplementary material has been provided by the authors to give readers additional information about their work.

© 2026 Finkelstein Y et al. *JAMA Network Open.*

eTable 1. *International Classification of Diseases, Tenth Edition* (*ICD-10*) Diagnosis Codes and *Anatomical Therapeutic Chemical* (*ATC*) Medication Codes Used in the Study

	Coding	Inclusion codes	Exclusion codes
Case definition			
Serious opioid intoxication events (SOE)	ICD-10	T40.0, T40.1, T40.2. T40.3, T40.4, T40.6	
Exposures			
Opioid prescription	ATC	N02A	
NSAID prescription	ATC	M01A	
Covariates			
Substance-related disorders	ICD-10	F55, F10-F19	F17
Mood and affective disorders	ICD-10	F30-F39	
Anxiety and stress disorders	ICD-10	F40-F48	F43
Schizophrenia or other psychotic disorders	ICD-10	F20-F29	
Other psychiatric disorders	ICD-10	F04-F99	F55, F10-F19, F20-F29, F30-F39, F40-F48
Suicidality	ICD-10	T14.91, R45.851	

© 2026 Finkelstein Y et al. *JAMA Network Open.*

eResults. Adjusted Odds Ratios for Serious Opioid Events With a Cutoff Age 10 Years

Children´s age < 10 years: Opioids vs. Unexposed: adjusted OR (95% CI) = 7.08 (4.87–10.30)

Children´s age < 10 years: Opioids vs. NSAIDs: adjusted OR (95% CI) = 4.11 (2.46–6.88)

Children´s age ≥ 10 years: Opioids vs. Unexposed: adjusted OR (95% CI) = 2.37 (1.98–2.84)

Children´s age ≥ 10 years: Opioids vs. NSAIDs: adjusted OR (95% CI) = 1.93 (1.54–2.41)

© 2026 Finkelstein Y et al. *JAMA Network Open.*

eTable 2. Sensitivity Analysis Incorporating Maternal Education as a Proxy for Socioeconomic Position in Models of Serious Opioid Events

Stratum	Category	Exposure	Adjusted OR* (95% CI)
Overall		Unexposed	Referent
		Opioid prescription	2.57 (2.18–3.03)
		NSAID prescription	Referent
		Opioid prescription	2.10 (1.71–2.58)
Child Sex	Girls	Unexposed	Referent
		Opioid prescription	3.65 (2.87–4.64)
		NSAID prescription	Referent
		Opioid prescription	3.70 (2.69–5.08)
	Boys	Unexposed	Referent
		Opioid prescription	1.93 (1.53–2.44)
		NSAID prescription	Referent
		Opioid prescription	1.36 (1.02–1.79)
Child Age	< 13 years	Unexposed	Referent
		Opioid prescription	6.26 (4.36–8.99)
		NSAID prescription	Referent
		Opioid prescription	3.61 (2.20–5.91)
	≥ 13 years	Unexposed	Referent
		Opioid prescription	2.12 (1.76–2.56)
		NSAID prescription	Referent
		Opioid prescription	1.83 (1.46–2.31)
Calendar period of SOE/index date	1995-2004	Unexposed	Referent
		Opioid prescription	1.90 (1.38–2.61)
		NSAID prescription	Referent
		Opioid prescription	1.55 (1.06–2.27)
	2005- 2014	Unexposed	Referent
		Opioid prescription	2.97 (2.36–3.74)

© 2026 Finkelstein Y et al. *JAMA Network Open.*

Stratum	Category	Exposure	Adjusted OR* (95% CI)
		NSAID prescription	Referent
		Opioid prescription	2.39 (1.78–3.20)
	2015-2022	Unexposed	Referent
		Opioid prescription	2.92 (2.05–4.16)
		NSAID prescription	Referent
		Opioid prescription	2.40 (1.52–3.79)

Abbreviations: CI – confidence interval; NSAIDs - non-steroidal anti-inflammatory drugs; OR – odds ratio; SOE - serious opioid events

*Odds ratios were controlled for the matching variables (i.e., age, sex, and calendar year) by study design and adjusted for child's mental health disorder, child's substance-related disorder, either parent with a mental health or substance misuse disorder, parental marital status, and maternal education as a proxy of socioeconomic position.

© 2026 Finkelstein Y et al. *JAMA Network Open.*
