# Supplementary material for: Intoxication in Children From Opioids Prescribed to Family Members
Source: JAMA Netw Open. 2026 Mar 26;9(3):e263515. doi: 10.1001/jamanetworkopen.2026.3515 (PMC13022737; doi:10.1001/jamanetworkopen.2026.3515)
Supplement: Supplement 2. — Data Sharing Statement [file jamanetwopen-e263515-s002.pdf]

Data Sharing Statement

Finkelstein. Intoxication in Children From Opioids Prescribed to Family Members. *JAMA Netw Open*. Published March 26, 2026. doi:10.1001/jamanetworkopen.2026.3515

Data
Data available: No
